# Spatiotemporal analysis of sandfly fauna (Diptera: Psychodidae) in an endemic area of visceral leishmaniasis at Pantanal, central South America

**DOI:** 10.1186/1756-3305-7-364

**Published:** 2014-08-15

**Authors:** Aline Etelvina Casaril, Neiva Zandonaide Nazario Monaco, Everton Falcão de Oliveira, Gabriel Utida Eguchi, Antonio Conceição Paranhos Filho, Luciana Escalante Pereira, Elisa Teruya Oshiro, Eunice Aparecida Bianchi Galati, Nathália Lopes Fontoura Mateus, Alessandra Gutierrez de Oliveira

**Affiliations:** Post Graduate Program in Infectious and Parasitary Diseases, Federal University of Mato Grosso do Sul, Campo Grande, MS Brazil; Laboratory of Parasitology/CCBS, Federal University of Mato Grosso do Sul, Mato Grosso do Sul, Campo Grande, MS Brazil; Center for Zoonosis Control, Health Secretariat of the Municipality of Corumbá, Corumbá, MS Brazil; Department of Epidemiology, School of Public Health, University of São Paulo, São Paulo, SP Brazil; Laboratory of Geotechnologies Applied to the Environment, Federal University of Mato Grosso do Sul, Mato Grosso do Sul, Campo Grande, MS Brazil; GIS Laboratory for Environmental Applications, Federal University of Mato Grosso do Sul, Mato Grosso do Sul, Corumbá, MS Brazil

**Keywords:** Sandfly vector, Leishmaniasis, Geotechnology

## Abstract

**Background:**

Environmental changes caused by urbanization can cause alterations in the ecology and behavior of sandflies and in the epidemiology of leishmaniasis. Geotechnological tools allow the analysis and recognition of spatiotemporal patterns by monitoring and mapping risk areas of this vector-borne disease. This study aims to describe the sandfly fauna in the municipality of Corumbá and to compare it with the data described in a three-year period from 1984 to 1986 by Galati. A further aim was to analyze the influence of environmental changes on the composition of the fauna.

**Methods:**

Captures were conducted weekly from April 2012 to March 2013, in intra and peridomicile areas with automatic light traps, from 6:00 pm to 6:00 am. The following indices were calculated for both periods analyzed: Standardized Index of Species Abundance (SISA), Shannon’s diversity index (H) and Pielou’s index (J). The Normalized Difference Vegetation Index (NDVI) was extracted from a remote sensing LANDSAT-5 image.

**Results:**

In total, 7,370 specimens (6,169 males and 1,201 females) were collected, distributed among 12 species. *Lutzomyia cruzi* was the most frequent species (93,79%) and the first in the ranking of standardized species abundance index in both studies. The dominance of the species *Lu. cruzi* in the neighborhoods of Maria Leite and Centro was demonstrated by the low equitability index. The neighborhood of Cristo Redentor had the greatest diversity of sandflies in the present study and the second greatest in the study performed by Galati et al. (Rev Saúde Pública 31:378–390, 1997). Analyzing the satellite images and the NDVI from 1984 and 2010, the largest amount of dense vegetation was found in the neighborhood of Cristo Redentor.

**Conclusions:**

It was, therefore, possible to show how changes caused due to urbanization have affected the density and distribution of *Lu. cruzi* and other species over time. Moreover, the data suggest that different populations of sandflies adapt in different ways according to environmental conditions and the adaptation does not necessarily depends on the presence of high vegetation cover.

## Background

The first autochthonous human case of visceral leishmaniasis (VL) in the Americas was diagnosed in 1911 using parasitological methods. The disease was reported in a man from the Porto Esperança district of the municipality of Corumbá in the state of Mato Grosso do Sul, Brazil [1]. Until 1995, VL remained restricted in this state to the municipalities of Corumbá and Ladário, with the subsequent occurrence of an expansion in the geographical distribution of the disease to the surrounding regions of Campo Grande and Três Lagoas [2]. The parasitosis continued its expansion and urbanization, reaching 56 of the 79 municipalities of the state. From 1994 to 2012, 260 cases of VL were confirmed in Corumbá, with another eight cases confirmed in 2013. Corumbá is currently classified as an area of intense transmission of *Leishmania infantum*[3].

The transmission of the parasite occurs during the blood meal of female sandflies from the genera *Phlebotomus* (Old World) and *Lutzomyia* (New World) infected with the protozoa *Leishmania*[4]*.* A total of 976 species or subspecies of sandflies have described throughout the world [5]. Approximately 260 have been reported in Brazil and 59 are reported for the state of Mato Grosso do Sul [6, 7]. *Lutzomyia longipalpis* has been identified as a major vector in most focal points of transmission in the Americas [8].

The absence of *Lu. longipalpis* in Corumbá together with the epidemiological evidence and the finding of *Lu. cruzi*[9, 10] and *Lu. forattinii*[11] naturally infected by *L. infantum* indicate the involvement of these two species as vectors of the parasite in the urban area. However, as *Lu. cruzi* presents higher densities and wider distribution than *Lu. forattinii*, it is considered as the major vector.

The impact of human actions on the dynamics of vector-borne diseases has been discussed in some studies [12]. Environmental changes caused by urbanization, such as disorganized land occupation toward peripheral areas with native vegetation, can cause alterations in the ecology and behavior of sandfly vectors. Thus, the fauna in a particular community may undergo changes that can lead to the loss of biodiversity and/or an increase in the number of species that are frequently in the peridomicile environment [13, 14].

Geotechnology resources, such as geographic information system and remote sensing, have recently been used to correlate the occurrence of diseases to the biogeographical data. These tools allow analysis and recognition of spatiotemporal patterns by monitoring and mapping risk areas of diseases, especially vector-borne diseases [15]. Therefore, comparative studies of fauna with the aid of geotechnology tools can enable us to determine whether urbanization and environmental changes caused by humans have altered the ecology and behavior of insect vectors.

The present study sought to investigate the current urban sandfly fauna in the municipality of Corumbá, comparing it with that identified in the three-year period from 1984 to 1986 by Galati [9] and to analyze the influence of environmental changes on the composition of the species.

## Methods

### Area and study site

**Figure 1:**
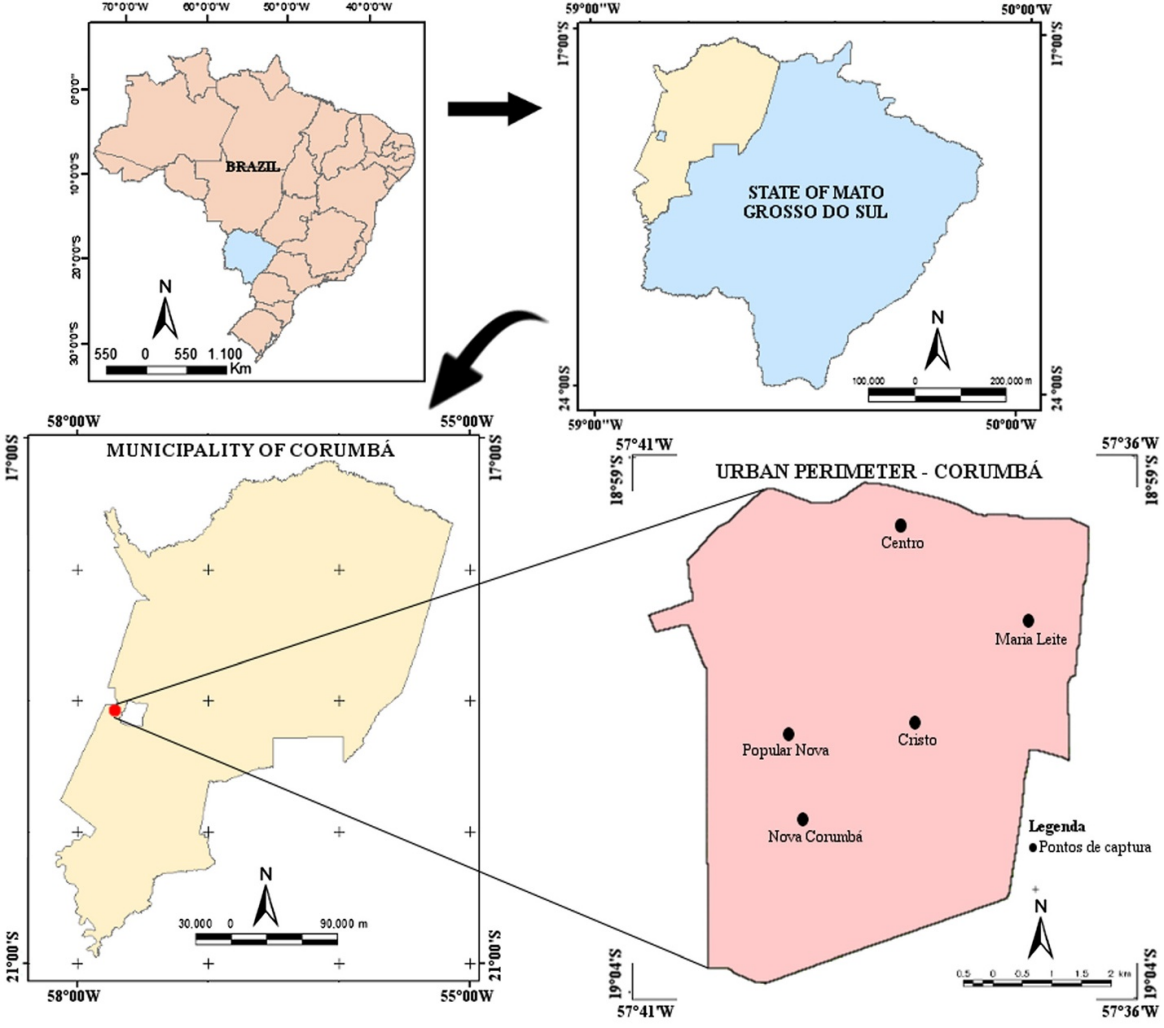
**Geographical location of study area: municipality of Corumbá, state of Mato Grosso do Sul, Brazil; 1: Centro; 2: Cristo Redentor; 3: Maria Leite; 4: Nova Corumbá; 5: Popular Nova.**

The municipality of Corumbá (18°59′44″ S and 57°19′36″ W; altitude: 116 m above sea level) is located in the northwestern portion of the state of Mato Grosso do Sul, Brazil, 415 km from the state capital, Campo Grande (Figure 1). It is considered the largest municipality in the state in terms of territorial extension, with an area of 64.962,720 km^2^. The urban area is situated on the bank of the Paraguay River and is dry bordered by Puerto Quijarro, Bolivia. According to the 2010 census of the Brazilian Institute of Geography and Statistics, the municipality has 103,703 inhabitants [16]. Soils in the municipality are predominantly lithic, shallow, medium texture to argillaceous and have low porosity. In some regions, gravel, pebbles, stones and associations with rocky outcrops are found, along with modifications in the landscape due to depositions of human origin (garbage dumps, metal refugeyards and rubble). The predominant vegetation is the typical savanna-like *cerrado* and the Pantanal wetland [17, 18]. The climate is tropical, megathermic, with a dry winter and rainy summer. The mean temperature is 25.1°C, with maximum and minimum annual temperature averages of 30.6 and 21.0°C, respectively. Mean relative humidity is 76.8% and annual rainfall is 1070 mm [19].

Five neighborhoods were chosen in the urban area of the municipality: Centro, Cristo Redentor, Maria Leite, Nova Corumbá and Popular Nova (Figure 1). In each of this neighborhood a dwelling was selected following two criteria: human cases of visceral leishmaniasis reported in 2011 and the presence of animal shelters as kennels and henhouses. Two of these neighborhoods (Centro and Cristo Redentor) were also investigated in the study conducted by Galati et al. [9].

The Centro neighborhood has the most human activity and its population has the highest socioeconomic status. It is located about 500 m from the Paraguay River and the residence chosen has a wide peridomicile area, with abundant vegetation and outbuildings (kennel and hen house).

Cristo Redentor is a peripheral neighborhood situated near hills covered with native vegetation. Many streets are unpaved and there are many vacant lots. A low-income population predominates in this area and the residence selected lies at the foothills, with the presence of poultry, dog kennels and large amounts of decomposing organic material.

Maria Leite is situated in the eastern region of the municipality near the forest area. The farm selected hosts social events and has the highest number of domestic animals (chickens, geese, Angolan chickens, birds and dogs).

Nova Corumbá is located in the southern region of the municipality and has a large number of vacant lots. The house selected is situated adjacent to remnants of native vegetation and a hen house. Several domestic animals (dogs, cats and birds) are present.

Popular Nova is located in the western region of the municipality. The house selected is small, close to a native forest and has a hen house and kennel.

### Captures

Captures were conducted weekly from April 2012 to March 2013. Light traps described by Falcão [20] were installed in the domicile and the peridomicile area from 6:00 pm to 6:00 am. The insects captured were identified based on Galati [21] and abbreviations of the genus followed the proposal put forth by Marcondes [22].

### Analysis

The following indices were calculated for both periods analyzed. The Standardized Index of Species Abundance (SISA) was used to determine the most abundant species according to spatial distribution, for which 1 corresponds to the most abundant species [23]. For the analysis of diversity, Shannon’s diversity index (H) was calculated and the measure of evenness or dominance of species was obtained using Pielou’s index (J) [24, 25]. Following the geo-referencing of the images, radiometric correction was performed, followed by arithmetic operations to calculate the Normalized Difference Vegetation Index (NDVI), which was comparatively evaluated through an analysis of satellite images. It is the index that measures the quantity of python mass and the density of vegetation [26]. These images were acquired from the National Institute for Spatial Research. Moreover, images from 1984 and 2010 obtained through Landsat 5 system were used for the multi-temporal analysis.

## Results

In total, 390 weekly captures were performed from April 2012 to March 2013. A total of 7,370 specimens of sandflies were collected, distributed among 12 species (Table 1). In the 754 samples obtained captures conducted by Galati et al. [9] from February 1984 to December 1986, 1,290 sandflies were collected, distributed among eight different species. Table 2 displays the number of sandflies captured in the urban areas of Corumbá in both periods.

**Table 1:** **Distribution of sandfly species by sex and ecotopes in the municipality of Corumbá, state of Mato Grosso do Sul, Brazil, April 2012 to March 2013 (n = 7,370)**

Species	Centro				Cristo Redentor				Maria Leite				Nova Corumbá				Popular Nova							%
	Intra		Peri		Intra		Peri		Intra		Peri		Intra		Peri		Intra		Peri		Total		Total	
	M	F	M	F	M	F	M	F	M	F	M	F	M	F	M	F	M	F	M	F	M	F		
*Br. brumpti*	-	-	-	-	-	-	-	-	-	-	-	-	1	-	1	-	-	-	-	-	2	-	2	0,03
*Ev. aldafalcaoae*	-	-	2	-	1	-	-	-	-	-	-	-	-	-	-	-	-	-	-	-	3	-	3	0,04
*Ev. cortelezzii*	-	-	-	-	-	1	-	-	-	-	-	-	-	-	-	2	-	-	-	-	-	3	3	0,04
*Ev. corumbaensis*	5	17	3	22	6	17	8	16	1	1	1	8	1	11	1	9	1	2	2	8	29	111	140	1,90
*Ev. sallesi*	-	1	1	1	-	1	-	-	-	-	-	-	-	-	-	-	-	-	-	-	1	3	4	0,05
*Ev. walkeri*	-	-	1	-	-	-	-	-	-	-	-	-	-	-	-	-	-	-	-	-	1	-	1	0,01
*Lu. cruzi*	1278	196	456	176	397	89	404	67	91	27	1070	88	493	71	1454	227	49	25	215	39	5907	1005	6.912	93,79
*Lu. forattinii*	3	1	10	3	56	25	86	11	2	1	4	1	15	4	19	13	-	-	3	3	198	62	260	3,53
*Mi. peresi*	-	1	-	-	12	6	3	3	-	-	-	-	-	1	2	3	-	-	-	2	17	16	33	0,45
*Mt. oliveirai*	-	-	-	-	4	-	5	-	-	-	-	-	-	-	1	-	-	-	-	-	10	-	10	0,14
*Pa. bigeniculata*	-	-	-	-	-	-	1	-	-	-	-	-	-	-	-	-	-	-	-	-	1	-	1	0,01
*Sc. sordellii*	-	-	-	-	-	1	-	-	-	-	-	-	-	-	-	-	-	-	-	-	-	1	1	0,01
**Total**	1286	216	473	202	476	140	507	97	94	29	1075	97	510	87	1478	254	50	27	220	52	6169	1201	7370	100

*Br: Brumptomyia; Ev: Evandromyia; Lu: Lutzomyia; Mi: Micropygomyia; Mt: Martinsmyia; Pa: Psathyromyia; Sc: Sciopemyia;* M: male; F: female.

**Table 2:** **Number of sandflies captured in the urban areas of Corumbá, state of Mato Grosso do Sul, Brazil, in two periods (Feb. 1984 to Dec. 1986 and Apr. 2012 to Mar. 2013)**

Species	1984/1986						2012/2013					
	M	%	F	%	Total	%	M	%	F	%	Total	%
*Br. brumpti*	1	0.11	1	0.26	2	0.16	2	0.03	-	-	2	0.03
*Ev. aldafalcaoae*	-	-	-	-	-	-	3	0.05	-	-	3	0.04
*Ev. cortelezzii*	-	-	-	-	-	-	-	-	3	0.25	3	0.04
*Ev. corumbaensis*	56	6.14	74	19.58	130	10.08	29	0.47	111	9.24	140	1.90
*Ev. sallesi*	3	0.33	1	0.26	4	0.31	1	0.02	3	0.25	4	0.05
*Ev. walkeri*	-	-	-	-	-	-	1	0.02	-	-	1	0.01
*Lu. cruzi*	634	69.51	196	51.85	830	64.34	5907	95.75	1005	83.68	6912	93.79
*Lu. forattinii*	121	13.27	50	13.23	171	13.25	198	3.20	62	5.16	260	3.53
*Mi. peresi*	34	3.73	24	6.35	58	4.49	17	0.28	16	1.33	33	0.45
*Mt. oliveirai*	1	0.11	0	0.00	1	0.08	10	0.16	-	-	10	0.14
*Pa. bigeniculata*	-	-	-	-	-	-	1	0.02	-	-	1	0.01
*Sc. sordellii*	62	6.80	32	8.47	94	7.29	-	-	1	0.09	1	0.01
**Total**	**912**	**100**	**378**	**100**	**1290**	**100**	**6169**	**100**	**1201**	**100**	**7370**	**100**

*Br*: *Brumptomyia*; *Ev*.: *Evandromyia*; *Lu*: *Lutzomyia*; *Mi*: *Micropygomyia*; *Mt*: *Martinsmyia*; *Pa*.: *Psathyromyia*; *Sc*.: *Sciopemyia*; M: male; F: female.

An analysis of Shannon’s Diversity Index (H) reveals a higher rate in the Dom Bosco neighborhood, followed by Cristo Redentor in the study conducted by Galati et al. [9]. In the present study, the greatest diversity of species was found in Cristo Redentor. Similar results were found in the analysis of Pielou’s index (J) (Table 3).

**Table 3:** **Shannon’s Index and (H) Pielou’s Index (J) for sandflies captured in urban areas of Corumbá, state of Mato Grosso do Sul, Brazil, in two periods (Feb. 1984 to Dec. 1986 and Apr. 2012 to Mar. 2013)**

Neighborhood	1984/1986		2012/2013	
	H	J	H	J
Centro	0.4288	0.2664	0.1752	0.0900
Cristo Redentor	1.1707	0.6534	0.7385	0.3207
Dom Bosco	1.6201	0.7791	-	-
Maria Leite	-	-	0.0862	0.0785
Nova Corumbá	-	-	0.1929	0.0991
Popular Nova	-	-	0.2830	0.2041

In both studies, the most frequent species were *Lutzomyia cruzi*, *Lutzomyia forattinii* and *Evandromyia corumbaensis*, which were also the most abundant species in 2012–2013 according to the SISA. From 1984 to 1986, the most abundant species were *Lu. cruzi*, *Ev. corumbaensis* and *Sciopemyia sordellii* (Table 4).

**Figure 2:**
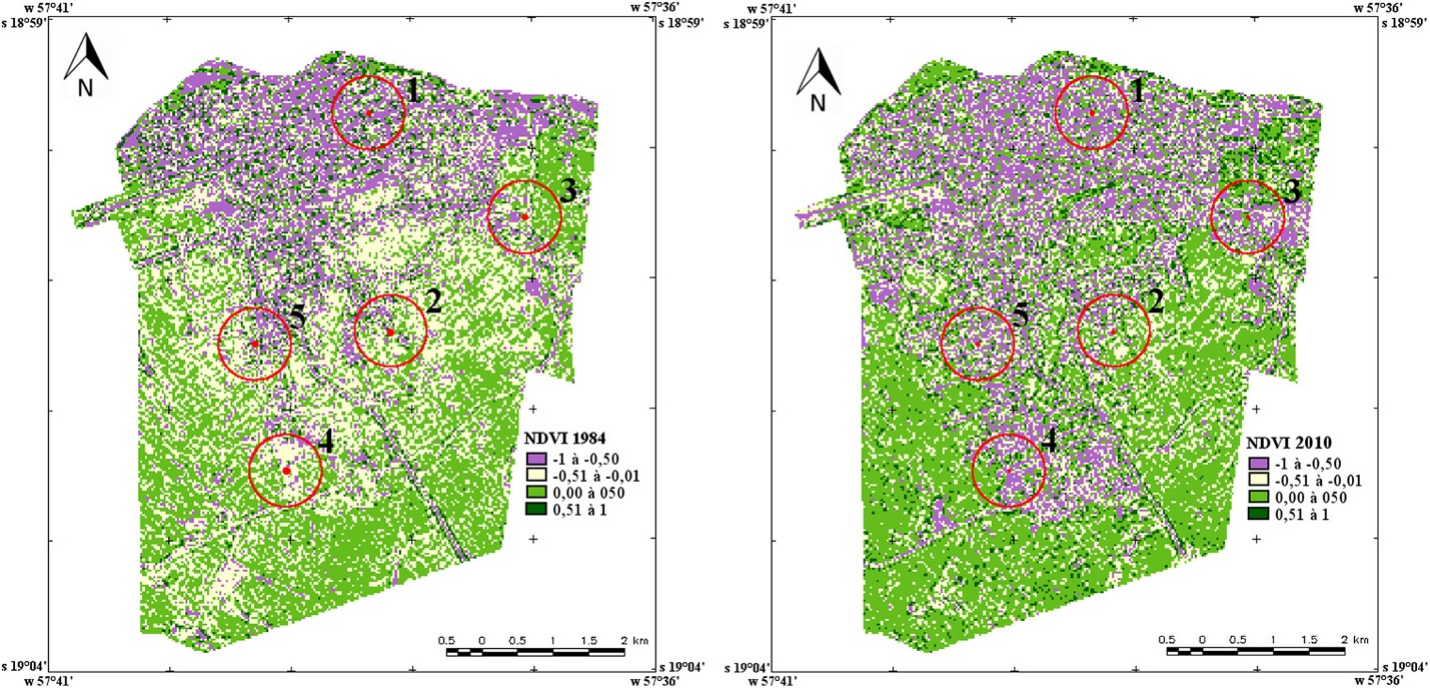
**LANDSAT 5 Image and Normalized Difference Vegetation Index (NDVI) for collection areas in Corumbá, state of Mato Grosso do Sul, Brazil, in two periods (1984 and 2010);1: Centro; 2: Cristo Redentor; 3: Maria Leite; 4: Nova Corumbá; 5: Popular Nova.**

Figure 2 displays the vegetation index (NDVI) from 1984 and 2010 at five capture points. Values closer to 1.00 denote a greater the amount of biomass. Values above 0.51 indicate the presence of forest vegetation and values below -0.50 represent minimal vegetation. The largest vegetation indices (NDVI: 0.51 to 1.00) occurred in the Cristo Redentor neighborhood.

**Table 4:** **Standardized Index of Species Abundance (SISA) of sandflies captured in Corumbá, state of Mato Grosso do Sul, Brazil, in two periods (Feb. 1984 to Dec. 1986 and Apr. 2012 to Mar. 2013)**

Species	1984/1986		2012/2013	
	SISA	Position	SISA	Position
*Br. brumpti*	0.13	5^th^	0.09	5^th^
*Ev. aldafalcaoae*	-	-	0.31	4^th^
*Ev. cortelezzii*	-	-	0.31	4^th^
*Ev. corumbaensis*	1.00	1^st^	1.00	1^st^
*Ev. sallesi*	0.44	4^th^	0.31	4^th^
*Ev. walkeri*	-	-	0.09	5^th^
*Lu. cruzi*	1.00	1^st^	1.00	1^st^
*Lu. forattinii*	0.96	2^nd^	1.00	1^st^
*Mi. peresi*	0.92	3^rd^	0.86	2^nd^
*Mt. oliveirai*	0.06	6^th^	0.42	3^rd^
*Pa. bigeniculata*	-	-	0.09	5^th^
*Sc. sordellii*	1.00	1^st^	0.09	5^th^

*Br: Brumptomyia; Ev: Evandromyia; Lu: Lutzomyia; Mi: Micropygomyia; Mt: Martisnmyia Pa: Psathyromyia; Sc: Sciopemyia.*

Table 5 displays the color composition of the collection points within a radius of 500 and 1000 meters. The buffers situated on the left of each column represent the quantification of the NDVI in each study period, while buffers on the right are composed of false colors and are only qualitative. A greater amount of red indicates greater biomass. Table 6 displays the statistical values for the 500-meter and 1000-meter vegetation buffers.

**Table 5:** **500-meter and 1000-meter vegetation buffers at collection points in Corumbá, state of Mato Grosso do Sul, Brazil, in two periods (1984 and 2010)**

500-m buffer		
Neighborhood/Year	1984	2010
Centro		
Cristo Redentor		
Maria Leite		
Nova Corumbá		
Popular Nova		
**1000-m buffer**		
**Neighborhood/Year**	**1984**	**2010**
Centro		
Cristo Redentor		
Maria Leite		
Nova Corumbá		
Popular Nova

**Table 6:** **Mean, standard deviation and variance of Normalized Difference Vegetation Index at sandfly collection sites with radii of 500 and 1000 meters; Corumbá, Mato Grosso do Sul, Brazil (1984 and 2010)**

Neighborhood	Mean		Standard deviation		Variance	
	1984	2010	1984	2010	1984	2010
**500-m buffer**						
Centro	80	75	69	72	4750	5189
Cristo	98	107	41	54	1659	2950
Maria Leite	102	90	46	68	2162	4650
Nova Corumbá	96	94	32	65	1010	4205
Popular Nova	87	92	48	68	2328	4691
**1000-m buffer**						
Centro	74	76	68	72	4631	5229
Cristo	97	109	39	51	1557	2648
Maria Leite	102	100	45	63	2046	3935
Nova Corumbá	102	104	34	60	1175	3639
Popular Nova	93	102	43	65	1847	4155

## Discussion

The diversity and distribution of sandfly species constitute essential knowledge regarding the epidemiological risks of leishmaniasis. The use of geotechnological tools in endemic areas allows monitoring the insect population and identifying increases in the density of focal points of dipteran vectors. Such data are important to the establishment of more effective control measures and the surveillance of this vector-borne disease [27, 28].

Comparing the fauna surveys conducted in Corumbá in the periods analyzed, Galati et al. [9] found eight species of sandflies, all of which were also found in the present study. However, *Ev. aldafalcoae*, *Ev. cortellezzii*, *Ev. walkeri* and *Pa. bigeniculata* had not been encountered by Galati et al. [9]. The present data expand knowledge on sandfly fauna in the municipality, since *Ev. walkeri* has not been previously reported. In contrast, the other three species mentioned were described in a study by Almeida et al. [8]. However, it should be noted that *Pa. bigeniculata,* which is considered a junior synonym of *Pa. shannoni* has been recently revalidated [29].

The increase in sandfly fauna is likely the result of urbanization. The hills of Corumbá are mainly composed of *cerrado* vegetation and have been intensely occupied, as demonstrated by the NDVI. Thus, humans can come into contact with new species of sandflies when invading areas of native vegetation and building homes in the surroundings.

The increase in the urban population of *Lu. cruzi* and the drastic reduction in the *Sc. sordellii* population are noteworthy. According to Rangel and Vilela [28], environmental changes caused by human actions, such as deforestation, fire, agricultural expansion, extractivism, rural settlements, mining, dams and hydroelectric plants, are capable of changing the ecology of some species of sandflies and *Leishmania* in Brazil, thereby altering the epidemiology of leishmaniasis. Some of these factors are found in Corumbá and they may explain the maintenance of the disease in the municipality, since residents live in close contact with elements of the epidemiological chain.

In the present study, the most urbanized areas (NDVI -1 to -0) exhibited a high frequency of *Lu. cruzi*. A number of studies also report the ability of *Lu. longipalpis* to occupy urban areas. The high frequency of *Lu. cruzi* in urban environments has previously been reported by Galati et al. [9] and Almeida et al. [8] in Corumbá (state of Mato Grosso do Sul) and by Missawa et al. [30] in Jaciara (state of Mato Grosso). However, no previous study has demonstrated this association using spatial data.

Initially, it was believed that *Lu. cruzi* was restricted to the municipalities of Bonito, Camapuã, Campo Grande, Corumbá and Ladário in the state of Mato Grosso do Sul. However, entomological studies have shown that this species also has wide geographical distribution in the state of Mato Grosso, where it has been reported in 25 municipalities [7, 9, 10, 31].

According to Missawa and Lima [31], the highest frequencies of *Lu. cruzi* are found in municipalities with areas of the Pantanal wetland and the savanna-like *cerrado*, indicating that these are the preferred environments of this species. This accounts for the high frequency of *Lu. cruzi* in Corumbá, as the municipality is located in the southern portion of the Pantanal region.

*Lu. cruzi* is morphologically similar to *Lu. longipalpis* and the two are considered sibling species belonging to a species complex [5]. Mangabeira [32] suggests that *Lu. cruzi* could be a regional variation of *Lu. longipalpis*. Examining the holotype of *Lu. cruzi*, Martins et al. [33] found that these species can be considered cryptic due to their similarities and because the females are morphologically indistinguishable. This similarity could explain the adaptation of *Lu. cruzi* to the urban environment, as previously reported for *Lu. longipalpis*.

The presence of a particular species in an ecotope is determined by its adaptation to the conditions, environmental resources and competitive interactions with other species [34]. As *Lu. cruzi* and *Lu. longipalpis* are phylogenetically very close, they must use the same ecological resources, resulting in the elimination of one species by competition. Almeida et al. [8] suggest that this species selection process could explain the absence of *Lu. longipalpis* in two municipalities studied in the state of Mato Grosso do Sul where *Lu. cruzi* was found.

*Sciopemyia sordellii* has been found in slightly modified environments, such as rural areas, forests, caves and caverns [9, 35, 36]. Thus, due to urbanization and the consequent reduction in biomass in the municipality of Corumbá, a decline of this species is noted when comparing the two periods. This fact confirms that each species reacts in a particular way to changes caused by humans.

*Lu. forattinii* was the second most frequent species in the present study and was found concomitantly in areas of occurrence of *Lu. cruzi. Lu. forattinii* is restricted to municipalities in Mato Grosso do Sul and has been found in Corumbá, Ladário and Anastácio [7]. Galati et al. [9] warned of the possibility that *Lu. forattinii* might also take part in the VL transmission chain in Corumbá. This assumption is justified by the fact that this species belongs to the same monophyletic group as *Lu. longipalpis* and *Lu. cruzi*; it also exhibits a high degree of density in Corumbá and is very anthropophilic [9].

To identify the role of *Lu. cruzi* and *Lu. forattinii* in the VL transmission chain in Corumbá, Pita-Pereira et al. [11] submitted female sandflies from the municipality to polymerase chain reaction analysis. The infection rate by *L. infantum* was 1.5% for *Lu. cruzi* and 0.7% for *Lu. forattinii*. This evidence reaffirms the inferences proposed by Galati et al. [9], reveals the epidemiological importance of these species in the region and underscores the need of further studies, such as investigations into vector competence [9].

The capture in a sympatric area of the three following species from the cortellezii complex is noteworthy: *Ev. sallesi*, *Ev. corumbaensis* and *Ev. cortelezzii*. The latter species was not encountered by Galati et al. [9]. Epidemiological significance has recently been attributed to this group, after *Ev. cortelezzii* and *Ev. sallesi* females were found naturally infected with parasites of the genus *Leishmania* in the states of Minas Gerais and Mato Grosso do Sul [37–39]. There are no records of these species biting humans and, although the scientific community has not implicated them as vectors for the transmission of *Leishmania* spp, the possibility that species are involved in wild or rural cycles of leishmaniasis transmission cannot be ruled out.

The male-to-female ratio of 5.14:1 demonstrates a significant predominance of males. This is in agreement with data described by Nunes et al. [40], who found a 5.7:1 ratio in Bonito, Almeida et al. [41] who found a 4.8:1 in Ponta Porã, and Oliveira et al. [42] who found a 2.95:1 in Campo Grande (all in the state of Mato Grosso do Sul). Galati et al. [9] has also found a larger proportion of males than females in Corumbá, but the 1.2:1 ratio is far lower than the one reported herein [9]. A higher proportion of males may be explained by normal male behavior of monitoring females for mating. Males are active in their pursuit of hosts and, upon finding them, release pheromones to attract females [43]. Another related fact is that females are hematophagous and seek refuge in shelters for digestion after feeding.

The high number of specimens collected in the present study may be explained by the presence of animal shelters on the peridomiciles selected, as sandflies are found in abundance on dogs and chickens [44]. Another relevant factor was the presence of vegetation, moist soil due to the disposal of domestic water, areas shaded by trees and the accumulation of organic matter on the ground (leaves, fallen fruits, feces of domestic animals and food scraps). These aspects mimic the natural habitats of these dipterans [45–47].

In this study, Falcão model [20] light traps were installed. These traps are based on the principle of light attraction and are employed to study the behavior of sandflies in forested and rural areas or in peridomicile and intradomicile environments. Despite their limitations, such as the interference of light from the urban environment and the greater attraction of males, these traps are more appropriate due to their low cost, small size and low nuisance to residents. Moreover, this type of trap achieves similar results when compared to the modified CDC traps used by Galati et al. [9].

The topology of Corumbá is characterized by flat areas among a set of residual hills. The Paraguay River is the northern limit of the urban area and the southern boundary is associated with Morraria Maciço de Urucum and correlated relief. The substrate of the hills surrounding the urban area is Precambrian, mainly composed of carbonates. The weathering of the rocks on the hills mainly produces a clay-like material that fills the valleys between the hills, often as residual material or colluvium and constitutes the substrate of the flat areas. The clay allows retention of soil moisture, which is important to the life cycle of the sandfly. Moreover, lesser human occupation is found at higher parts of the slope, where the vegetation is preserved. The vegetated hills are bordered by more intensively occupied areas. This topology leads to close contact between the population and vegetation.

Regarding the ecotypes evaluated in Corumbá, the neighborhood of Cristo Redentor had the greatest diversity of sandflies in the present study and the second greatest in the study by Galati et al. [9]. This may be explained by the location of the residences analyzed in the study, which were close to native vegetation, including the foothills. The natural habitat of sandflies is characterized by little variation in temperature and humidity, as these insects are very sensitive to desiccation. Thus, small changes in these factors in the microhabitat can alter the population dynamics of the insects [48].

Analyzing the satellite images and NDVI from 1984 and 2010, the largest amount of dense vegetation was found in the neighborhood of Cristo Redentor within both 500 and 1000 meters. This vegetation provides better conditions for the development of immature specimens and winged sandflies and also provides better shelter and food. In these periods, both the vegetation and urbanization in this neighborhood grew toward the field area. In areas without human activities, the regeneration of the hill forests occurred. This fact confirms the importance of planning urban growth to preserve fauna and flora as well as the importance of housing construction at a minimum distance of 400 to 500 m from woods to avoid the maintenance of leishmaniasis [49].

The dominance of the species *Lu. cruzi* in the neighborhoods of Maria Leite and Centro was demonstrated by the low equitability index. In the two study periods, the Centro neighborhood had the greatest absence of biomass, with little change between periods, as urban occupation and a quiet heterogeneous area was already well established in 1984. The neighborhood of Maria Leite underwent the greatest loss of vegetation due to urban expansion in this area.

Analyzing satellite images from 1984, the neighborhoods of Popular and Nova Corumbá had a similar NDVI and more homogeneous areas, with the predominance of vegetation and small urban areas. In the image from 2010, an increase in population and urbanization was noted (NDVI:-1 to -0.50) in these neighborhoods. However, the amount of dense vegetation also increased. This is due to the fact that urban occupation took place in field areas (NDVI -0.51 to -0.01), while vegetation also developed in this same area with no human settlement. Therefore, housing construction near forest areas provided more contact with a diversity of sandfly species.

The highest abundance of *Lu. cruzi* was found in the peridomicile environment of the neighborhood of Nova Corumbá. This finding may be explained by the high NDVI (0.51 to 1.00) and the presence of the henhouse. The demand for feeding is a behavioral aspect that affects the reproduction and population density of sandflies. Chickens (*Gallus gallus domesticus*) are refractory to infection by protozoa of the genus *Leishmania* and are therefore not reservoirs of the parasite. However, these animals are important in the epidemiological chain of VL as a source of attraction and feeding for female sandflies [50–52].

Few studies have combined fauna research with geotechnology [53]. In Feira de Santana, state of Bahia, Carneiro et al. [54] demonstrated that locations with a spatial distribution pattern of *Lu. longipalpis* and reported cases of VL had low NDVI values and vegetation, likely due to human actions. In Campo Grande, state of Mato Grosso do Sul, Oliveira et al. [55] found that the density of *Lu. longipalpis* was not affected by the heterogeneity of the habitat, but rather by its complexity.

## Conclusions

The urbanization and adaptation of vectors have promoted the geographical spread of leishmaniasis in Brazil. Therefore, fauna studies are of considerable importance [56], as they provide data for the establishment of more effective control measures. In the present study, geotechnological tools allowed the identification and quantification of the diversity of vegetation using the NDVI. It was therefore possible to show that the decrease in vegetation cover caused by urbanization could have affected the density and distribution of *Lu. cruzi* and other species over time. Moreover, the data suggest that different populations of sandflies adapt in different ways in accordance with the environmental conditions and do not necessarily depend on the presence of high vegetation cover. Thus, the species in question must be very eclectic and non-demanding, as demonstrated by their adaptation to the urbanization process and consequent decrease in vegetation cover.

The disorderly occupation of the human population toward hill areas covered by native vegetation, as occurred in the municipality studied, places humans in close contact with wild species of sandflies. This demonstrates the need for continual, intensive entomological surveillance for the prevention and control of visceral leishmaniasis in Corumbá.

## References

[1] Migone LE (1913). Un caso de Kalazar a Assuncion (Paraguay). Bull Soc Exot Path.

[2] Antonialli SAC, Torres TG, Paranhos-Filho AC, Tolezano JE (2007). Spatial analysis of American Visceral Leishmaniasis in Mato Grosso do Sul State, Central Brazil. J Infect.

[3] Mato Grosso do Sul: *Governo do Estado de Mato Grosso do Sul. Secretaria de Saúde de Saúde do Estado Estadual de Vigilância Epidemiológica Coordenadoria. Estadual de Zoonoses Gerência. Informe epidemiológico das leishmanioses nº 4/2012*. http://www.saude.ms.gov.br/controle/ShowFile.php?id=123472

[4] Ready PD (2010). Leishmaniasis emergence in Europe. Euro Surveill.

[5] Galati EAB (2013). Phlebotominae (Diptera, Psychodidae): classificação, morfologia, terminologia, identificação de adultos. Bioecologia e identificação de Phlebotominae. Departamento de Epidemiologia, Faculdade de Saúde Pública.

[6] Shimabukuro PHF, Galati EAB (2011). Checklist dos Phlebotominae (Diptera, Psychodidae) do estado de São Paulo, Brasil, com comentários sobre sua distribuição geográfica. Biota Neotrop.

[7] Lainson R, Rangel E (2005). *Lutzomyia longipalpis* and the eco-epidemiology of American visceral leishmaniasis, with particular reference to Brazil - a review. Mem Inst Oswaldo Cruz.

[8] Almeida PS, Nascimento JC, Ferreira AD, Minzão LD, Portes F, Miranda AM, Faccenda O, Andrade-Filho JD (2010). Espécies de flebotomíneos (Diptera, Psychodidae) coletadas em ambiente urbano em municípios com transmissão de Leishmaniose Visceral do Estado de Mato Grosso do Sul, Brasil. Rev Bras Entomol.

[9] Galati EAB, Nunes VLB, Rego JFA, Oshiro ET, Chang MR (1997). Estudo de flebotomíneos (Diptera: Psychodidae) em foco de leishmaniose visceral em Mato Grosso do Sul, Brasil. Rev Saude Publica.

[10] Santos SO, Arias J, Ribeiro AA, Hoffmann MP, Freitas RA, Malacco MAF (1998). Incrimination of *Lutzomyia cruzi* as a vector of American Visceral Leishmaniasis. Med Vet Entomol.

[11] Pita-Pereira D, Cardoso MAB, Alves CR, Brazil RP, Britto C (2008). Detection of natural infection in *Lutzomyia cruzi* and *Lutzomyia forattinii* (Diptera: Psychodidae: Phlebotominae) by *Leishmania infantum chagasi* in an endemic area of visceral leishmaniasis in Brazil using a PCR multiplex assay. Acta Trop.

[12] Morse SS (1995). Factors in the emergence of infectious diseases. Emerg Infect Dis.

[13] Gratz NG (1999). Emerging and resurging vector-borne diseases. Annu Rev Entomol.

[14] Bradley CA, Altizer S (2006). Urbanization and the ecology of wildlife diseases. Trends Ecol Evol.

[15] Barcellos C, Monteiro AMV, Corvalán C, Gurgel HC, Carvalho MS, Artaxo P, Hacon S, Ragoni V (2009). Mudanças climáticas e ambientais e as doenças infecciosas: cenários e incertezas para o Brasil. Epidemiol Serv Saúde.

[16] IBGE - INSTITUTO BRASILEIRO DE GEOGRAFIA E ESTATÍSTICA. IBGE@Cidades.2010. Corumbá, http://www.ibge.gov.br/cidadesat/xtras/perfil.php?codmun=500320&search=mato-grosso-do-sulCorumbá

[17] Pott VJ, Pott A, Universidade Federal do Paraná/IBAMA (1985). Flórula ruderal da cidade de Corumbá, MS. Congresso Nacional de Botânica, Curitiba.

[18] Cardoso EL, Oliveira H, Amaral JAM, Ker JC, Pereira NR, Santos RD, Tosto SG, Spera ST, Carvalho-Junior W, SILVA JSV (2000). Pedologia. Zoneamento ambiental. Borda Oeste do Pantanal. Maciço do Urucum e Adjacências.

[19] Soriano BMA (1997). Caracterização climática de Corumbá, MS.

[20] Falcão AR (1981). Um novo modelo de armadilha luminosa de sucção para pequenos insetos. Mem Inst Oswaldo Cruz.

[21] Galati EAB, RANGEL E, LAINSON R (2003). Morfologia e taxonomia: classificação de Phlebotominae. Flebotomíneos do Brasil.

[22] Marcondes CB (2007). A proposal of generic and subgeneric abbreviations of phlebotomine sandflies (Diptera: Psychodidae: Phlebotominae) of the world. Entomol News.

[23] Roberts DR, Hsi BP (1979). An index of species abundance for use with mosquito surveillance data. Environ Entomol.

[24] Deane LM, Deane MP, Ferreira-Neto JA, Almeida FB (1971). On the transmission of Simian Malaria in Brazil. Rev Inst Med Trop Sao Paulo.

[25] Hayek LAC, Buzas MA (1997). Surveying natural populations.

[26] PONZONI FJ, SHIMABUKURO YE (2007). Sensoriamento remoto no estudo da vegetação.

[27] Ashford RW (2000). The leishmaniases as emerging and reemerging zoonoses. Int J Parasitol.

[28] Rangel EF, Vilela ML (2008). *Lutzomyia longipalpis* (Diptera, Psychodidae, Phlebotominae) and urbanization of visceral leishmaniasis in Brazil. Cad Saúde Pública.

[29] Sabio PB, Andrade AJ, Galati EAB (2014). Assessment of the taxonomic status of some species included in the Shannoni Complex, with the description of a new species of Psathyromyia (Diptera: Psychodidae: Phlebotominae). J Med Entomol.

[30] Missawa NA, Veloso MAE, Maciel GBML, Michalsky EM, Dias ES (2011). Evidência de transmissão de leishmaniose visceral por *Lutzomyia cruzi* no município de Jaciara, Estado de Mato Grosso, Brasil. Rev Soc Bras Med Trop.

[31] Missawa NA, Lima GBM (2006). Distribuição Espacial de *Lutzomyia longipalpis* (Lutz & Neiva, 1912) e *Lutzomyia cruzi* (Mangabeira, 1938) no Estado de Mato Grosso. Rev Soc Bras Med Trop.

[32] Mangabeira-Filho O (1969). Sobre a sistemática e biologia dos Phlebotomus do Ceará. Rev Bras Mal Doenç Trop.

[33] Martins AV, Falcão AL, Silva JE, Dias ES (1984). Nota sobre *Lutzomyia (Lutzomyia) cruzi* (Mangabeira, 1938) com a descrição da fêmea (Diptera: Psychodidae: Phlebotominae). Mem Inst Oswaldo Cruz.

[34] Ricklefs RE (2003). A economia da natureza.

[35] Alves VR, Freitas RA, Santos FL, Barrett TV (2011). Diversity of sandflies (Psychodidae: Phlebotominae) captured in sandstone caves from Central Amazonia, Brazil. Mem Inst Oswaldo Cruz.

[36] Oliveira AG, Galati EAB, Oliveira O, Oliveira GRO, Espindola IAC, Dorval MEC, Brazil RP (2006). Abundance of *Luztomyia longipalpis* (Díptera: Psychodidae: Phlebotominae) and urban transmission of visceral leishmaniasis in Campo Grande, state of Mato Grosso do Sul, Brazil. Mem Inst Oswaldo Cruz.

[37] Andrade ARO, Dorval MEMC, Andrade SMO, Marques A, Lima-Jr MSC, Silva BAK, Andreotti R (2011). First report of natural infection of phlebotomines for *Leishmania (Leishmania) chagasi* captured in Ponta Porã, on the border between Brazil and Paraguay. Asian Pac J Trop Dis.

[38] Carvalho GML, Andrade-Filho JD, Falcão AL, Rocha-Lima AC, Gontijo CM (2008). Naturally infected *Lutzomyia* sand flies in a *Leishmania*-endemic area of Brazil. Vector Borne Zoonotic Dis.

[39] Saraiva L, Carvalho GM, Gontijo CM, Quaresma PF, Lima AC, Falcão AL, Andrade-Filho JD (2009). Natural infection of *Lutzomyia neivai* and *Lutzomyia sallesi* (Diptera: Psychodidae) by *Leishmania infantum chagasi* in Brazil. J Med Entomol.

[40] Nunes VLB, Galati EAB, Cardozo C, Rocca MEG, Andrade ARO, Santos MFC, Aquino RB, Rosa D (2008). Estudo de flebotomíneos (Diptera, Psychodidae) em área urbana do município de Bonito, Mato Grosso do Sul, Brasil. Rev Bras Entomol.

[41] Almeida OS, Minzão ER, Minzão LD, Silva SR, Ferreira AD, Faccenda O, Andrade-Filho JD (2010). Aspectos ecológicos de flebotomíneos (Diptera: Psychodidae) em área urbana do município de Ponta Porã, Estado de Mato Grosso do Sul. Rev Soc Bras Med Trop.

[42] Oliveira AG, Galati EAB, Fernandes CE, Dorval MEC, Brazil RP (2012). Ecological aspects of phlebotomines (Diptera: Psychodidae) in endemic area of visceral leishmaniasis, Campo Grande, State of Mato Grosso do Sul, Brazil. J Med Entomol.

[43] Jones TM, Hamilton JGC (1998). A role for pheromones in mate choice in a lekking sandfly. Anim Behav.

[44] Ximenes MFFM, Souza MF, Castellón EG (1999). Density of sand flies (Diptera: Psychodidae) in domestic and wild animal shelters in an area of visceral leishmaniasis in the state of Rio Grande do Norte, Brazil. Mem Inst Oswaldo Cruz.

[45] Leonardi MVC, Silveira TGV, Alves WA, Maia-Elkoury ANS, Membrive UA, Membrive NA, Rodrigues G, Reis N, Zanzarini PD, Ishikawa E, Teodoro U (2006). Leishmaniose tegumentar americana e canina no município de Mariluz, Estado do Paraná, Brasil. Cad Saúde Pública.

[46] Massafera R, Silva AM, Carvalho AP, Santos DR, Galati EAB, Teodoro U (2005). Fauna de flebotomíneos do município de Bandeirantes, no Estado do Paraná. Rev Saude Publica.

[47] Andrade ARO, Nunes VLB, Galati EAB, Arruda CCP, Santos MFC, Aquino RB (2009). Epidemiological study on leishmaniasis in an area of environmental tourism and ecotourism, State of Mato Grosso do Sul, 2006–2007. Rev Soc Bras Med Trop.

[48] Dias ES, França-Silva JC, Silva JC, Monteiro EM, Paula KM, Gonçalves CM, Barata RA (2007). Flebotomíneos (Diptera: Psychodidae) de um foco de leishmaniose tegumentar no Estado de Minas Gerais. Rev Soc Bras Med Trop.

[49] Brasil. Ministério da Saúde (2007). Manual de vigilância da leishmaniose tegumentar americana.

[50] Alexander B, Carvalho RL, Mccallum H, Pereira MH (2002). Role of the domestic chicken (*Gallus gallus*) in the epidemiology of urban visceral leishmaniasis in Brazil. Emerg Infect Dis.

[51] Roach JC, Glusman G, Rowen L, Kaur A, Purcell MK, Smith KD, Hood LE, Aderem A (2005). The evolution of vertebrate Toll-like receptors. Proc Natl Acad Sci.

[52] Oliveira AG, Marassá AM, Consales CA, Dorval MEC, Fernandes CE, Oliveira GR, Brazil RP, Galati EAB (2008). Observations on the feeding habits of *Lutzomyia longipalpis* (Lutz & Neiva, 1912) (Diptera: Psychodidae: Phlebotominae) in Campo Grande, an endemic area of visceral leishmaniasis in Mato Grosso do Sul, Brazil. Acta Trop.

[53] Andrade ARO, Silva BAK, Cristaldo G, Andrade SMO, Paranhos-Filho AC, Ribeiro A, Santos MFC, Andreotti R (2014). Spatial distribution and environmental factors associated to phlebotomine fauna in a border area of transmission of visceral leishmaniasis in Mato Grosso do Sul, Brazil. Parasit Vectors.

[54] Carneiro D, Bavia ME, Rocha W, Lobão J, Madureira-Filho J, Oliveira JB, Silva CE, Barbosa MG, Rios R (2004). Identificação de áreas de risco para leishmaniose visceral americana, através de estudos epidemiológicos e sensoriamento remoto orbital, em Feira de Santana, Bahia, Brasil (2000–2002). Rev Baiana Saúde Pública.

[55] Oliveira EF, Silva EA, Fernandes CES, Paranhos-Filho AC, Gamarra RM, Ribeiro AA, Brazil RP, Oliveira AG (2012). Biotic factors and occurrence of *Lutzomyia longipalpis* in endemic area of visceral leishmaniasis, Mato Grosso do Sul, Brazil. Mem Inst Oswaldo Cruz.

[56] Rêgo FD, Shimabukuro PHF, Quaresma PF, Coelho IR, Tonelli GB, Silva KMS, Barata RA, Dias ES, Gontijo CMF (2014). Ecological aspects of the Phlebotominae fauna (Diptera: Psychodidae) in the Xakriabá Indigenous Reserve, Brazil. Parasit Vectors.

